# Chinstrap penguin population genetic structure: one or more populations along the Southern Ocean?

**DOI:** 10.1186/s12862-018-1207-0

**Published:** 2018-06-13

**Authors:** Isidora Mura-Jornet, Carolina Pimentel, Gisele P. M. Dantas, Maria Virginia Petry, Daniel González-Acuña, Andrés Barbosa, Andrew D. Lowther, Kit M. Kovacs, Elie Poulin, Juliana A. Vianna

**Affiliations:** 1 Departamento de Ecosistemas y Medio Ambiente, Facultad de Agronomía e Ingeniería Forestal, Pontificia Universidad Católica de Chile, Av. Vicuña Mackenna 4860, Macul, Santiago, Chile; 2 Instituto de Ecología y Biodiversidad, Departamento de Ciencias Ecológicas, Facultad de Ciencias, Universidad de Chile, Las Palmeras 3425, Ñuñoa, Santiago, Chile; 3 Pontifícia Universidade Católica de Minas Gerais, PPG in Biology of Vertebrate Av, Dom Jose Gaspar, 500, prédio 41, Belo Horizonte, 30535901 Brasil; 4 Laboratório de Ornitologia e Animais Marinhos, Universidade do Vale do Rio dos Sinos, Av. Unisinos, São Leopoldo, RS 950 Brazil; 5 Departamento de Ciencias Pecuarias, Facultad de Ciencias Veterinarias, Universidad de Concepción, Av. Vicente Méndez 595, 3780000 Chillán, CP Chile; 6 Departamento de Ecología Evolutiva, Museo Nacional de Ciencias Naturales, CSIC, C/José Gutiérrez Abascal, 2, 28006 Madrid, Spain; 7 Norwegian Polar Institute, Hjalmar Johansensgata, Tromsø, Norway

**Keywords:** Seabirds, *Pygoscelis antarcticus*, Dispersal, Gene flow, Microsatellites, D-loop, Sex-biased, Antarctica

## Abstract

**Background:**

Historical factors, demography, reproduction and dispersal are crucial in determining the genetic structure of seabirds. In the Antarctic marine environment, penguins are a major component of the avian biomass, dominant predators and important bioindicators of ecological change. Populations of chinstrap penguins have decreased in nearly all their breeding sites, and their range is expanding throughout the Antarctic Peninsula. Population genetic structure of this species has been studied in some colonies, but not between breeding colonies in the Antarctic Peninsula or at the species’ easternmost breeding colony (Bouvetøya).

**Results:**

Connectivity, sex-biased dispersal, diversity, genetic structure and demographic history were studied using 12 microsatellite *loci* and a mitochondrial DNA region (HVRI) in 12 breeding colonies in the South Shetland Islands (SSI) and the Western Antarctic Peninsula (WAP), and one previously unstudied sub-Antarctic island, 3600 km away from the WAP (Bouvetøya). High genetic diversity, evidence of female bias-dispersal and a sign of population expansion after the last glacial maximum around 10,000 mya were detected. Limited population genetic structure and lack of isolation by distance throughout the region were found, along with no differentiation between the WAP and Bouvetøya (overall microsatellite *F*_*ST*_ = 0.002*, p =* 0.273*;* mtDNA *F*_*ST*_ *=* − 0.004*, p =* 0*.*766), indicating long distance dispersal. Therefore, genetic assignment tests could not assign individuals to their population(s) of origin. The most differentiated location was Georges Point, one of the southernmost breeding colonies of this species in the WAP.

**Conclusions:**

The subtle differentiation found may be explained by some combination of low natal philopatric behavior, high rates of dispersal and/or generally high mobility among colonies of chinstrap penguins compared to other *Pygoscelis* species.

**Electronic supplementary material:**

The online version of this article (10.1186/s12862-018-1207-0) contains supplementary material, which is available to authorized users.

## Background

Investigating population genetic structure is important for understanding evolutionary processes [1], and establishing conservation actions for species [2]. Genetic structure is mainly determined by four processes: demography, historical factors, mating system and dispersal [3, 4]. Population demography is influenced by biotic and abiotic factors that promote variability across a species’ range. Habitat suitability, topographical barriers, resource availability and quality, and interaction with other organisms are drivers that can lead to genetic differentiation between populations [5]. Historical factors, such as the Last Glacial Maximum (LGM) and the expansion or isolation of species in different refugia also affect evolutionary processes [6, 7]. Life-history traits such as mating systems can affect genetic structure at both biparentally and maternally inherited markers, sometimes differently [1]. Finally, migratory patterns also influence genetic structure by affecting spatial patterns, species’ ranges, and environmental adaptation of the species [8]. The predisposition of an individual to return to its natal colony throughout its reproductive lifetime is known as philopatry [9]. In most birds, females have a greater tendency to leave their natal groups and disperse larger distances than males [9, 10]. These sex biases in dispersal are important to investigate, to understand its evolution [11].

Population genetic structure is typically investigated using indirect methods, such as inferring gene flow levels among colonies [12]. As levels of gene flow increase towards panmixia, the power to statistically detect distinct populations using clustering algorithms decreases [13, 14]. Additionally, population size has implications for genetic differentiation, as larger populations are more robust to the effects of genetic drift than smaller ones [15]. Thus, considering these processes, three broadly different patterns of population genetic structure can be observed: 1) absence of both genetic structure and differentiation among populations, 2) significant genetic structure, but no geographic pattern to explain it, or 3) significant genetic and geographic structured populations [5].

In the Southern Ocean, penguins are a major component of the avian biomass [16], dominant predators [17], and bioindicators of ecosystem changes [18, 19]. In the South Shetland Islands (SSI) and the Western Antarctic Peninsula (WAP), three species of *Pygoscelis* penguins breed sympatrically: Adélie (*Pygoscelis adelie*), gentoo (*P. papua*) and chinstrap (*P. antarcticus*) [20]. Population genetic structure of these penguins has recently been well documented. Microsatellite and mitochondrial data on Adélie penguins have revealed a lack of genetic differentiation between colonies around the Antarctic continent, and a sign of population expansion after the LGM [21–23]. In contrast, genetic markers employed for gentoo penguins’ analyses have revealed significant population genetic structure in Antarctica, and also evidenced divergent lineages between Antarctica and each sub-Antarctic colony studied [6, 24]. This is explained by the presence of a physical barrier (Antarctic Polar Front) and large geographical distances [24].

Unlike Adélies and gentoos, almost the entire breeding distribution of chinstrap penguins is restricted to the Antarctic Peninsula (up to approximately 64° S) and the South Shetland, South Orkney, and South Sandwich Islands in the Scotia Sea region [20, 25–29]. Additionally, small breeding populations are described on South Georgia, Bouvetøya, Heard and the Balleny Islands [20, 27]. The non-breeding range of the chinstrap penguin is extensive, with large dispersal being reported*.* To exemplify, Trivelpiece et al. [30] demonstrated through satellite telemetry that penguins could migrate from the South Shetland Islands to the South Orkney and South Sandwich Islands, 800 and 1300 km away, respectively. Biuw et al. [31] described a migration of 3600 km from Bouvetøya to the South Sandwich Islands for a single pre-moulting adult chinstrap penguin. Although all three *Pygoscelis* species show some degree of natal philopatry, chinstrap penguins are the least philopatric of the genus [32, 33]. At fine geographical scales, this species appears to show weak or even no significant population structure, with no isolation by distance [22, 34, 35]. No sex-bias has been detected for these birds using microsatellite *loci* [35], although the authors reported test values consistent with female bias dispersal. Currently, chinstrap penguins are listed as of Least Concern on the IUCN’s Red List of Threatened Species [36]. However, there have been reports of continuous declines at nearly all breeding sites of this species [33, 34, 37–46].

Population genetic structure of chinstrap penguins has been studied in some parts of their range [22, 34, 35]. However, this species remains the least studied of the *Pygoscelis* penguins, and the connectivity between breeding colonies in the WAP, or between the WAP and the easternmost breeding colony in the species’ distribution (Bouvetøya), is still unknown. Population declines reported in numerous colonies highlight the importance of investigating the connectivity of breeding colonies in terms of source and sink population dynamics, and other genetic effects these reductions might have. Indeed, in the context of conservation biology, the proper identification of population genetic structure is crucial [2]. Thus, to investigate this, we used 12 microsatellite markers and mitochondrial DNA Hypervariable Region I (HVRI) sequences of chinstrap penguins from 13 different locations to: (1) investigate the demographic history following the LGM, (2) describe patterns of distribution of genetic diversity and population structure, (3) quantify levels of connectivity among colonies in the WAP and the easternmost limit of the species’ distribution at Bouvetøya, and (4) evaluate levels of sex-biased dispersal. We proposed two hypotheses: (a) lack of or reduced genetic structure among breeding colonies of chinstrap penguins in Antarctica, as observed in *P. antarcticus* in a few locations and in another species of the genera with similar ecological features (*P. adeliae*), and (b) strong genetic structure between Antarctica and Bouvetøya, due to isolation explained by large geographical distances.

## Methods

### Field sampling and DNA extraction

**Fig. 1:**
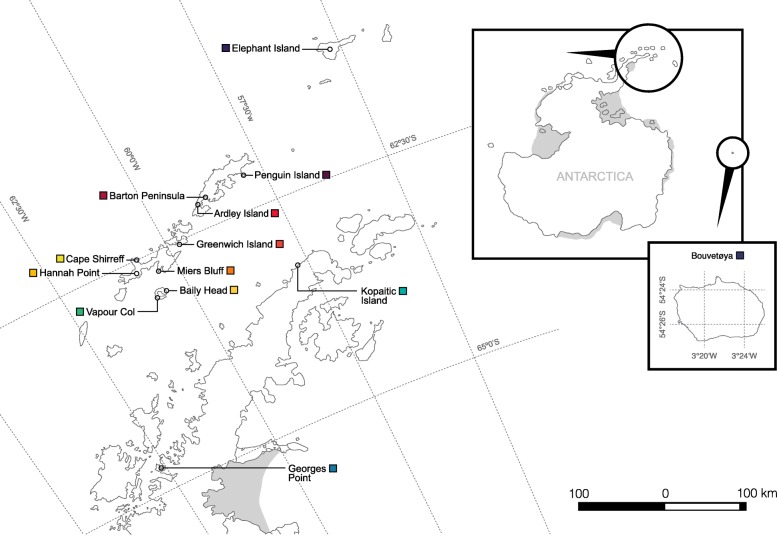
Chinstrap penguin sampled sites during this study (total *n* = 251)

Between January and February of 2009 and 2016 (plus Miers Bluff in 2003), a total of 251 blood samples from chinstrap penguins were collected at 13 sites, including 10 locations in the South Shetland Islands (*n* = 183), two in the Antarctic Peninsula (*n* = 45) and Bouvetøya (*n* = 23) (Fig. 1 and Table 1). To avoid disturbance within the breeding colonies, adult penguins were captured using hand-held nets while entering the water. Each individual was stained with bromophenol blue to avoid re-sampling. Up to 1 mL of blood was obtained from brachial or medial metatarsal veins using a 23 G needle, and stored in 96% ethanol. All procedures were done following an accepted restraining method for penguins [47].

**Table 1:** Summary of chinstrap penguin samples used, genetic diversity indices and neutrality test results

							HVRI					Microsatellite *loci*				
Region	Colony (abbreviation)	Coordinates		N	H	S	Hd	*π*	*∏*	D	Fs	N	A	Ho	He	F_*IS*_
WAP – SSI	Elephant Island (EI)	61°13’S	55°21’W	17	13	13	0.96	0.01	2.35	−1.47	**− 10.1**	17	5.55	0.62	0.62	0.003
	Penguin Island (PI)	62°06’S	57°56’W	17	14	19	0.98	0.01	3.43	**−1.54**	**−9.47**	19	5.91	0.70	0.67	−0.053
	Barton Peninsula (BP)	62°14’S	58°46’W	22	19	18	0.98	0.01	3.37	−1.17	**−17.4**	29	6.18	0.57	0.62	0.081
	Ardley Island (AI)	62°13’S	58°56’W	12	10	13	0.97	0.01	3.26	−1.03	**−5.45**	14	5.27	0.60	0.66	0.096
	Greenwich Island (GI)	62°31’S	59°47’W	14	12	16	0.98	0.01	2.97	**−1.68**	**−8.57**	14	5.18	0.61	0.64	0.048
	Miers Bluff (MB)	62°43’S	60°26’W	9	8	10	0.97	0.01	2.89	−1.00	**−4.47**	11	5.00	0.61	0.62	0.019
	Hannah Point (HP)	62°39’S	54°36’W	20	14	17	0.95	0.01	3.13	−1.30	**−8.19**	25	5.91	0.64	0.64	0.001
	Cape Shirreff (CS)	62°28’S	60°48’W	22	18	19	0.97	0.01	2.92	**−1.63**	**−16.4**	30	6.46	0.61	0.64	0.039
	Baily Head (BH)	62°58’S	60°30’W	8	7	9	0.96	0.01	2.60	−1.21	**−3.67**	9	5.10	0.70	0.71	0.011
	Vapour Col (VC)	63°00’S	60°44’W	13	13	15	1	0.01	3.44	−1.21	**−11.9**	15	5.46	0.67	0.65	−0.025
WAP - AP	Kopaitic Island (KI)	63°19’S	57°55’W	26	26	32	1	0.01	4.40	**−1.77**	**−25.7**	30	6.36	0.63	0.64	0.028
	Georges Point (GP)	64°40’S	62°39’W	9	9	14	1	0.01	3.72	−1.33	**−6.00**	15	5.82	0.64	0.64	−0.003
SAI	Bouvetøya (BI)	54°26’S	3°23′E	18	16	18	1	0.01	3.44	−1.32	**−13.1**	23	5.73	0.65	0.65	−0.001
	Total	61°10’S	55°00’W	207	119	55	0.98	0.01	3.32	**−1.92**	**−25.9**	251	5.69	0.63	0.65	0.003

Bold values are significantly different from zero after the FDR correction (*p* < 0.05 for D and *p* < 0.01 for Fs)

Geographical regions (SAI: Sub-Antarctic Islands; WAP: West Antarctic Peninsula; SSI: South Shetland Islands; AP: Antarctic Peninsula), information of localities (with abbreviations), coordinates, number of samples used from each locality for each marker (HVRI and 11 microsatellite *loci*), genetic diversity indices’ results (N: sample size; H: number of haplotypes; S: number of polymorphic sites; Hd: haplotype diversity; π: nucleotide diversity; ∏: pairwise difference, A: mean number of alleles per *locus*; Ho: mean observed heterozygosity; He: mean expected heterozygosity and F_*IS*_: inbreeding coefficient) and neutrality tests (D: Tajima’*D* test, Fs: Fu’s Fs test)

Total genomic DNA was extracted using a salt protocol [48], modified as follows: a lysis buffer based on TNE 1X, Tris-HCl pH 7.8 and SDS 25% in place of Tris-HCl pH 8.0, EDTA and SDS 20%. Additionally, 10 M ammonium acetate was used instead of NaCl, and tubes spun down for 20 min at 14, 000 rpm. After the extraction, DNA samples were stored in TE Buffer (Tris-EDTA; 10 mM Tris base, 0.1 mM EDTA) at − 20 °C or − 80 °C.

### Amplification, sequencing and genotyping protocols

A 305 base pair (bp) of the mitochondrial DNA (mtDNA) HVRI was amplified using forward primer Forw2 (5’-ACAGTACGAGATAAGTCATGGTTCC-3′) or L-tRNA^Glu^ (5′-CCCGCTTGGCTTYTCTCCAAGGTC-3), and reverse primer AH530 (5′- CTGATTTCACGTGAGGAGACCG-3′) [49]. The PCR conditions and amplification cycles were done following Peña et al. [6]. The mtDNA PCR products were purified and Sanger sequenced bi-directionally in Macrogen Inc. (Seoul, South Korea). All mtDNA sequences were deposited in GenBank accession numbers: MF966819 – MF966902 and MH025646 – MH025759.

Genetic diversity and population differentiation were examined at 12 tetranucleotide microsatellite *loci* (AP-3, AP-19, AP-26, AP-61, AP-78, AP-85, AP-90, CP-6, CP-25, GP-6, GP-15 and GP-36) isolated from the genome of three species of *Pygoscelis* penguin sequenced by NGS (Next Generation Sequencing), as part of another study [50]. Forward primers were synthesized using 5′-end-M13 tail-labelled fluorophores with one of three dyes (6-FAM, HEX, or NED; Applied Biosystems) to adjust simultaneous genotyping at multiple *loci* with overlapping size ranges. Protocols applied for primer’s sequences, PCR conditions, and amplification cycles for microsatellite *loci* were those described in Vianna et al. [50]. DNA samples were separated by electrophoresis through a 2% agarose gel, run for 0.5 h at 300 V. Genotyping of the obtained PCR products were performed at Macrogen Inc. (Seoul, South Korea). All PCRs were conducted in an Applied Biosystem machine, and the mixtures contained 10–100 ng of genomic DNA. The microsatellite genotypes were assigned using GeneMarker® v.1.75 (Softgenetics LLC™) software for allele size identification.

### Gene diversity

The mtDNA sequences (HVRI) were aligned and edited according to the chromatogram utilizing Sequencher v.5.1 (Gene Codes, Ann Arbor, MI, USA). Polymorphic sites (S), number of haplotypes (h), haplotype diversity (Hd), average number of differences between pairs of sequences (∏), and nucleotide diversity (π) were estimated with Arlequin v.3.5.1.2 [51], applying 10,000 permutations. To create an mtDNA haplotype network, the sequence alignment was used to create a maximum parsimony tree using MEGA7 [52]. For this analysis, the program determined K2 + G + I (Kimura 2-parameter + gamma + invariable sites) as the best substitution model with a gamma parameter of 0.54. The maximum parsimony tree generated subsequently by MEGA7 and the sequence alignment were used to generate a haplotype network with Haplotype Viewer [53].

For all microsatellite data, PGDSpider v.2.1.0.1 software was used as an automated data conversion tool [54]. For these data sets, the presence of null alleles or potential genotyping errors were evaluated using Micro-Checker v.2.2.3 [55]. Arlequin v.3.5.1.2 [51] was used to study genetic diversity within samples from each chinstrap penguin colony, calculate the mean number of alleles per *locus*, and evaluate the observed (Ho) and expected heterozygosities (He). Expectations for Hardy-Weinberg equilibrium (HWE) were estimated as deviation of the Wright’s *F*_*IS*_ index and these were tested for each *locus*, for all *loci*, and for each population utilizing randomization procedures using 10,000 permutations with GENETIX v.4.05.2 [56]. To test the presence of linkage disequilibrium, the same program was applied with a likelihood-ratio test and the empirical distribution generated by 10,000 permutations. Corrections for multiple testing were made using the False Discovery Rate (FDR) [57, 58].

### Population genetic structure and isolation by distance

Arlequin v.3.5.1.2 [51] was utilized to calculate *F*_*ST*_ and *Ф*_*ST*_ between pairwise populations on microsatellite and mtDNA sequence data using 10,000 permutations. *P* values were corrected with the FDR method for multiple tests [57, 58]. Employing microsatellite *loci*, isolation by distance was evaluated by means of the Adegenet package in R [59]. For this, Adegenet uses a Mantel test between a matrix of genetic distances, and a matrix of geographical distances [60]. Google Earth (Google, v.7.1.8.3036) was used to calculate the shortest geographical distance by sea between locations.

To determine the most likely number of clusters (*K*), multilocus genotypes were analyzed through Bayesian clustering methods implemented in STRUCTURE v2.3.4 [61], BAPS v.6.0 [62] and GENELAND v.3.1 [63]. The software STRUCTURE v2.3.4 was run using different models assuming (ad)mixture, (un)correlated allele frequencies both with and without a priori specification of sample locations [61, 64]. The models were run with the likely number of populations (*K*) set from 1 to 13. For each *K*, the model was run 10 times with a burn-in length of 100,000 iterations followed by 1,000,000 Markov Chain Monte Carlo (MCMC) subsequent iterations. The optimum number of clusters was inferred by deriving the posterior probability of *K* (LnP(D)) from each independent run. As the Δ*K* method of Evanno’s does not allow *K* = 1 to be tested [65], this method was employed when *K* was higher than one for log-likelihood using STRUCTURE HARVESTER [66]. To align multiple replicates of files produced by STRUCTURE, CLUMPP v1.1.2 (CLUster Matching and Permutation Program) [67] was applied. Results generated by the genetic clustering program were visualized through DISTRUCT v1.1 [68].

A Bayesian Analysis of Population Structure (BAPS v6.0) was performed using a combination of analytical and stochastic methods, based on molecular markers and geographical sampling [62]. Calculations were performed over 10,000 iterations with both spatial and non-spatial, and both a mixture and an admixture model, with the maximum number of populations possible set to 13.

An analysis of spatial structure using the R package GENELAND v3.1 was carried out to determine the most likely number of populations and to assign individuals to population clusters. This program is based on an algorithm which includes not only genotypes, but also the geographical location of all individuals to estimate the number of groups and delineate their spatial boundaries [69]. Analyses were performed under the spatial model assuming both correlated and uncorrelated allele frequency. The correlated frequency model, in comparison with the uncorrelated frequency model, might be more capable of detecting subtle differentiations. However, it could also be more sensitive to departure from model assumptions (as presence of isolation-by-distances), and more prone to algorithm instabilities [69]. Ten independent MCMC simulations were run allowing the number of populations to vary between 1 and 13, with the following parameters: 1,000,000 MCMC iterations with a thinning of 100, a maximum rate of Poisson processes fixed to 500 and a maximum number of nuclei in the Poisson-Voroni tessellation fixed to 300. The best-supported *K* value was determined based on the highest averaged maximum likelihood score of the models.

Additionally, a Discriminant Analysis of Principal Components (DAPC) was carried out to determine the number of clusters of genetically related individuals, using a non-Bayesian approach. DAPC uses sequential *K*-means and model selection to identify genetic clusters [70]. The Adegenet package in R [59] was used, retaining all principal components.

To assign or exclude individual colonies as being the origins of individuals based on genotype data, assignment testing of microsatellite *loci* was done using GENECLASS2 v.2.0.h [71]. Two separate analyses were performed: one employed the likelihood method based on allele frequencies [72], and the other used the Bayesian method approach [73]. The probability that each individual was assigned to a candidate population was estimated using a Monte Carlo resampling method (number of simulated individuals = 10,000; type I error = 0.01) [74]. The same program and parameters were also applied for the detection of first-generation migrants.

### Demographic history

To evaluate deviations from Wright-Fisher equilibrium, two neutrality tests were applied: Tajima [75] and Fu [76]. Both tests were performed in Arlequin v.3.5.1.3 [51]. The historical demographic changes were inferred and reconstructed with a Bayesian approach using BEAST v.1.8 [77] and Tracer v.1.5.0 [78] programs. The coalescence model elected was Bayesian Skyline plot and the molecular clock utilized was Lognormal relaxed clock (uncorrelated). The best fit nucleotide substitution model determined with Jmodeltest v2.1.10 [79] was HKY + G + I (Hasegawa-Kishino-Yano + gamma + invariant sites). The mutational rate used was 0.55 s/s/millions of years [80]. The MCMC chain length was 50,000,000 sampled every 1000 generations.

### Sex determination and sex-biased dispersal

For molecular sex identification, a region of the Chromosome-helicase-DNA binding protein (CHD1) gene was amplified, with primer pair 2550F/2718R [81]. PCRs were carried out in 25 μL volume containing 10–100 ng genomic DNA, 1X reaction buffer, 0.5 μM of each primer, 1.5 mM MgCl_2_, 100 *μ*M dNTPs and 0.7 U *Taq* DNA polymerase (Invitrogen Life Technologies). The reactions’ conditions were as follows: an initial denaturing step at 94 °C for 5 min; followed by 45 cycles at 94 °C for 30 s, 46 °C for 45 s, and 72 °C for 25 s; and a final extension step at 72 °C for 5 min. All reactions were conducted on an Applied Biosystem machine. The amplification products were separated on 2% agarose gel for approximately 1 h at 150 V and visualized with GelRed® under UV light.

Using microsatellite data of the sex-identified individuals by molecular techniques, sex-biased dispersal was evaluated with FSTAT v.2.9.3.2 [82]. Two hypotheses were tested: a one-tailed test was done assuming males as the most philopatric group, since dispersal is female-biased in most birds [9]. Next, a two-sided test under the assumption of no differences between male and female dispersion in chinstrap penguins [35]. For both tests, differences in the inbreeding coefficient (F_*IS*_), fixation index (F_*ST*_), relatedness between individuals (*r*), mean Assignment Index (*mAIc*) and variance of Assignment Indices (*vAIc*) between sexes were calculated. F_*ST*_*, r* and *mAIc* were expected to be lower in the sex that disperses most, whereas F_*IS*_ and *vAIc* were expected to be higher [11]. The *p* values of each test were estimated using 10,000 randomizations.

## Results

### Genetic diversity

For mtDNA HVRI results, high genetic diversity was found in all locations. A total of 119 haplotypes (*n* = 207, S = 55) were found, along with high haplotype diversity (*Hd =* 0.98, n = 207) ranging from 0.95 (HP) to 1 (VC, KI, GP and BI), and low nucleotide diversity (*π =* 0.01, Table 1). The number of haplotypes ranged from 7 (BH) to 26 (KI), while polymorphic sites ranged from 9 (BH) to 32 (KI).

For microsatellite data, only one *locus* was monomorphic (GP-6), so it was not used in further analyses. The remaining 11 microsatellite *loci* were polymorphic for all populations, except for AP-3 at Baily Head (BH) (Additional file 1: Table S1). The inbreeding coefficient (F_*IS*_) was low in all populations, and no significant *p-*values were found, indicating no significant heterozygote excess or deficiencies. Therefore, deviations from HWE were not detected in populations at the 11 *loci* (Table 1). Linkage disequilibrium of each pair of *loci* was not detected within or among populations. For microsatellite markers, overall allele numbers *per locus* varied between three (*locus* AP-3) and 12 (*locus* GP-15), with an average of 5.69 alleles for all sample sites. The expected heterozygosity ranged from 0.62 (Elephant Island, Barton Peninsula and Miers Bluff) to 0.71 (BH), with an average of 0.65. The observed heterozygosity exhibits a similar level of variation with an average of 0.63 over all locations. Values of allelic richness ranged between 5.00 to 6.46 per sample site. *Locus*-by-*locus* allelic richness and diversity measures for each sample location are shown in the Additional file 1: Table S1.

### Population genetic structure and isolation by distance

**Fig. 2:**
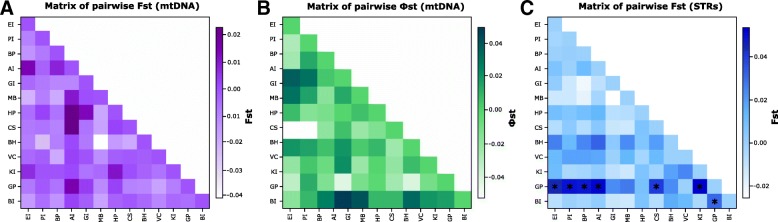
Distance matrices pairwise. **a**) pairwise F_*ST*_ values from mtDNA (HVRI), **b**) pairwise *Φ*_*ST*_ values from mtDNA (HVRI), and **c**) pairwise F_*ST*_ microsatellite values (STRs, 11 *loci*). Each cell of the heat plot is color-coded, illustrating relative differences. Darker colors indicate higher levels of genetic differences and white, lower. The asterisks indicate significant F_*ST*_ values

For pairwise values using mtDNA data, no significant genetic differentiation was found between any pairwise locations (Fig. 2a, b, and Additional file 2: Table S2 and Additional file 3: Table S3). Notably, there was also an absence of population genetic structure between the WAP and Bouvetøya (F_*ST =*_ − 0.004, *p* = 0.766) (Additional file 2: Table S2 and Additional file 3: Table S3).

For microsatellite *loci*, the F_*ST*_ values were generally not significant (Fig. 2c). Seven of 78 pairwise F_*ST*_ comparisons were significantly different, all corresponding to the southernmost locality of this study: Georges Point (GP). However, statistically significant F_*ST*_ values varied from 0.031 to 0.054, indicating a weak differentiation between GP and other colonies (Additional file 4: Table S4). GP F_*ST*_ values differed significantly from seven of the 13 northernmost studied sites (EI, PI, BP, AI, CS, KI and BI, Fig. 2c). Mantel’s testing did not detect isolation by distance in microsatellite data (*r* = 0.05, *p* = 0.40). Although studies have questioned the performance of the Mantel test [83, 84], it can be an effective approach if it is used cautiously [85].

To identify the number of populations among the 13 locations, four approaches were used with microsatellite *loci,* yielding different optimal numbers of clusters. Using the mean log-likelihood in STRUCTURE, the analysis inferred that the number of populations (*K*) was one, for seven of the eight tested different model assumptions. Only one model selected *K* = 3, therefore it was also evaluated by Evanno’s method, which suggested *K* = 2. Nonetheless, when analyzing individual assignment plots, no group could be identified (See Additional file 5: Figure S1). In the BAPS analysis, the inferred number of populations was *K* = 1 when the spatial model was applied, despite whether mixture or admixture models were performed. On the other hand, when a non-spatial model was run, the optimal number of clusters was *K* = 7, but without any geographical relation (Additional file 6: Figure S2). For GENELAND, the variation of estimated number of groups depended on whether the uncorrelated or correlated frequency model was used, although in both models the 10 runs consistency converged on a single *K* value. When employing the uncorrelated model, the inferred number of populations *K* was one. Contrastingly, GENELAND estimated *K* = 3 clusters for chinstrap penguins when the correlated allele frequency model was employed. These clusters corresponded to three distinct populations: (1) Kopaitic Island, (2) Georges Point, and (3) northern WAP locations and Bouvetøya (Additional file 7: Figure S3). Additionally, in the pairwise F_*ST*_ comparison, GENELAND also identified Georges Point as the most differentiated breeding colony, however, the probabilities of cluster membership were very low (< 0.5). Models and estimated number of populations (*K*) for all Bayesian programs used are summarized in Table 2. The final approach, DAPC, estimated the optimal number of clusters to *K* = 6, however, they were geographically meaningless and overlapped extensively (Additional file 8: Figure S4). Although some analyses suggested clusters larger than one, the graphic results did not show any consistent group. Finally, for estimation of dispersal patterns, assignment tests were only successful for assigning 13.9% of the individuals (assignment threshold of 0.05) to the proper colony, and low values were again observed for the first-migrant generation, revealing high gene flow among all sampled colonies (Additional file 9: Table S5).

**Table 2:** Bayesian clustering analyses and different models used to infer the optimal number of population (*K*)

Bayesian clustering software	Model use	Inferred number of cluster (*K*)
BAPS	Spatial, with mixture model	*K* = 1
	Spatial, with admixture model	*K* = 1
	Non-spatial model, with admixture model	*K* = 7
GENELAND	Spatial model, with uncorrelated allele frequency	*K* = 1
	Spatial model, with correlated allele frequency	*K* = 3
STRUCTURE	Admixture, with correlated allele frequency, using location information	*K* = 1
	Admixture, with independent allele frequency, using location information	*K* = 1
	Admixture, with correlated allele frequency, no location information supplied	*K* = 1
	Admixture, with independent allele frequency, no location information supplied	*K* = 1
	No admixture, with correlated allele frequency, using location information	*K* = 1
	No admixture, with independent allele frequency, using location information	*K* = 1
	No admixture, with correlated allele frequency, no location information supplied	*K* = 1
	No admixture, with independent allele frequency, no location information supplied	*K* = 3, K = 2 *

*Inferred number of cluster using Evanno’s method

### Demographic history

**Fig. 3:**
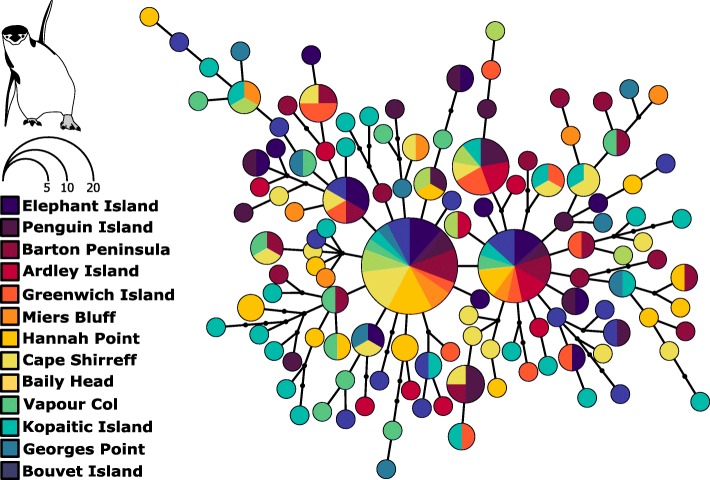
Haplotype network based on the mtDNA (HVRI) haplotypes according to sampling localities. Size of circles is proportional to haplotype frequency

The haplotype network analysis showed high genetic diversity, lack of divergent lineages and a star-like topology, suggesting population expansion (Fig. 3). A sign of population expansion was also observed for all studied locations using a Bayesian approach (Additional file 10: Figure S5); the historical time for this expansion was around 10,000 years ago. This is supported by the negative and significant values of the Tajima test (D = − 1.92, *p* < 0.05, Table 1) and Fu (Fs = − 25.9, *p* < 0.001, Table 1) for the species, and for the majority of the locations for Fu’Fs.

### Sex-biased dispersal

Sex of 196 individuals was determined: 93 females and 103 males. The majority of tests do not support sex-biased dispersal, however, the mean assignment index test revealed a significant difference between males and females for one-tailed and two-sided tests, that may indicate females as the dispersing sex in chinstrap penguins (Table 3).

**Table 3:** Sex-biased dispersal in chinstrap penguins. One-tailed test results, their corresponding p-values and the number (N) of females and males used for the analyses

				Assignment indices		
	N	*F*_*IS*_	*F*_*ST*_	Relatedness	Mean	Variance
Females	93	0.002	0.002	0.145	−0.509	9.56
Males	103	0.042	0.97	0.171	0.459	10.43
p-value		0.111	0.291	0.397	**0.029**	0.70

Bold values are significantly different from zero after the FDR correction (p < 0.05)

## Discussion

Chinstrap penguins throughout the 13 colonies studied herein, showed high levels of genetic diversity, low levels of genetic structure between study sites and no isolation by distance. Results also suggest female biased-dispersal and a sign of population expansion since the Last Glacial Maximum.

Although chinstrap penguin populations have decreased dramatically over the last four decades, high genetic diversity for both mtDNA and microsatellites were found in all studied colonies. This could be the result of a historically large population size or simply a result of the currently large population (7,5 million pairs) [25], combined with high levels of gene flow between colonies. This study’s finding of high genetic diversity is consistent with previous reports for chinstrap penguin colonies from the WAP, South Orkney and South Sandwich Islands, using microsatellite and mtDNA data [22, 35]. High genetic diversity has also been documented through mtDNA from gentoo penguins [6, 22, 24, 86] and microsatellite markers from Adélie penguins [21]. Additionally, investigations in other penguin species have also demonstrated high genetic diversity, such as in rockhopper [87], magellanic [88] and Humboldt penguins [89].

Using mtDNA (HVRI) from four breeding sites, Clucas et al. [22] found weak differentiation between colonies of chinstrap penguins from the WAP, South Shetland and South Orkney Islands in relation to the South Sandwich Islands. In this current study, no significant genetic differentiation was found in breeding colonies from the WAP and South Shetland Islands using the same marker. Most clustering data analyses suggests only one genetic group for chinstrap penguins. However, the lack of consensus reached for a few microsatellite analyses (Table 2) could be explained due to that the accuracy of Bayesian analyses commonly diminishes when levels of genetic differentiation among populations decreases, performing better with F_*ST*_ > 0.05 [14]. Microsatellite *loci* showed absence or reduced population structure among chinstrap penguins breeding in the WAP. Interestingly, absence of structure between the WAP and Bouvetøya was observed (Additional file 5: Figure S1 and Additional file 6: Figure S2, and Additional file 4: Table S4). These results complement and confirm previous genetic investigations that have found little (if any) population structure between chinstrap penguins in Antarctica with the use of microsatellites. For example, limited genetic variation was found among colonies from the WAP and archipelagos within the Scotia Arc. Nonetheless, limited numbers of breeding colonies (only two in the WAP, and two in the Scotia Arc) were studied [35]. Weak genetic differences and high level of gene flow between two colonies from the South Shetland Islands were also found using amplified fragment length polymorphism (AFLP) analyses, but in populations located within a short distance of 32 km [34]. The limited genetic structure found in the present study is likely the result of recurrent and long-distance migration of individuals between sample sites, supported by the inability of assignment tests to successfully place individuals in their exact populations of origin. The slight levels of genetic differentiation reported between Georges Point and the northernmost studied locations coincide with one of the southernmost distributions of chinstrap penguins along the Antarctic Peninsula. Thus, the incipient differentiation may be explained by a founder effect from the northernmost colonies to the south. Although the source colony is expected to present higher genetic diversity values than the newer ones [90], the genetic diversity indices found here were similar in all sample sites. A similar pattern was observed in the trumpeter finch (*Bucanetes githagineus*) in peripheral populations [91]. Another observed pattern that could support the hypothesis of chinstrap penguins colonizing new breeding habitats is that they are currently expanding their range southward along the Antarctic Peninsula [92]. Numerous studies have reported the presence of small numbers of chinstrap penguins south of their natural breeding range [93–95]. During field work conducted in January 2017, this was also observed: two breeding pairs on Waterboat Point (Gabriel González Videla base; 64°49’S, 62°51’W), a single individual surrounded by gentoos on Doumer Island (Yelcho base; 64°65’S, 63°35’W) and another single chinstrap surrounded by Adélies on Avian Island (67°46’S, 68°54’W) (Additional file 11: Figure S6). This may suggest that chinstraps tend to prospect other colonies and breeding habitats far away from their colony of origin, similar to that observed in king penguins [96].

Dispersal has significant effects on population size (growth or reduction), species’ persistence and genetics [97]. In most birds, dispersal tends to be female-biased, however, male-biased natal dispersal has been reported for Adélie penguins [98]. In contrast, the first study which compared connectivity between males and females through genetic tools in chinstrap penguins, reported several value test results (females with higher F_*IS*_, negative *mAIc* and higher *vAIC*) pointing towards a female-biased dispersal, though none of the indexes were significant [35]. In the current study, our data suggests that females are the dispersing sex and males are the philopatric sex (Table 3). However, this should be considered with caution, as most of the sex biased tests were not conclusive. Some studies mention that penguins are not always philopatric [32, 33]. Natal philopatry evince that individuals are likely to have low rates of movement between colonies [21]. However, only a proportion of all individuals are faithful to one locality [9], and a small number of migrants could homogenize population structure easily [99]. Indeed, Adélie penguins, which exhibit strong natal philopatry, do not show strong genetic difference among colonies, potentially due to interaction between large effective population sizes in combination with some dispersal [21]. Evolutionary reasonings for sex-biased dispersal are inbreeding avoidance and evasion of intersexual competition [10]. Philopatry has several benefits, such as development of antipredator strategies, social facilitation and spatial heterogeneity of breeding and foraging habitats [32]. However, stressful environmental conditions (such as extensive sea ice or obstruction to usual migration patterns) may be driving an increase in dispersion rates, leading penguin species to have less philopatric behaviour than previously thought [100].

Finally, chinstrap penguins also show a signature of population expansion after the Last Glacial Maximum, similar to that detected for other *Pygoscelis* penguins in the WAP [6, 22, 24]. Potentially, the LGM may have contracted the populations at lower latitudes such as the Scotia Arc and Bouvetøya, maintaining the large population size here observed, followed by a reexpansion about 10,000 years ago. This scenario of recolonization may have contributed to the absence of population genetic structure in Antarctica, and between Antarctica and Bouvetøya. A pattern of historical divergent lineages has been described between gentoo penguin colonies throughout sub-Antarctic islands with shorter geographical distances [24] than those observed for chinstrap penguins.

## Conclusions

Most of the data analyses suggests a single large population of chinstrap penguins throughout the Southern Ocean, with minimal population structure in the WAP region, and absence of genetic differentiations between the WAP and a sub-Antarctic island located 3600 km away. Georges Point, one of the southernmost breeding colonies of chinstrap penguin in the Antarctic Peninsula, was the most differentiated of all. The lack of genetic structure among distant reproductive colonies of chinstrap penguins in the Southern Ocean may be due to different factors, such as a historical large population size making it unyielding to drift, long-range gene flow between breeding colonies, stressful environmental conditions forcing penguins to increase dispersion rates and/or post-LGM recolonization between WAP and Bouvetøya.

## Additional files

Additional file 1: **Figure S1.** Plot of assignment probabilities from STRUCTURE. A vertical bar represents an individual, and colors represent the different clusters found. All plots were generated via running 10 replicates. Figures show the optimal number of clusters for no admixture model, with independent allele frequency and no location information supplied using A) Posterior probability of *K* (LnP(D)) and B) Evanno’s method. (DOCX 20 kb)

Additional file 2: **Figure S2.** Plot of assignment probabilities from BAPS. Vertical lines represent each individual and the color refers to clusters found through this analysis. A) spatial with both, mixture and admixture models (*K* = 1) and B) non-spatial admixture model (*K* = 7). (DOCX 16 kb)

Additional file 3: **Figure S3.** Posterior probabilities of population membership from the spatial model with correlated allele frequencies’ model. Lighter colors indicate higher probabilities of population membership. Three genetic clusters were identified using GENELAND. Left: Kopaitic Island, middle: Georges Point, and right: northern WAP locations and Bouvetøya. (DOCX 16 kb)

Additional file 4: **Figure S4.** Discriminant Analysis of Principal Components (DAPC). The six genetic clusters identified by Adegenet are shown in different colors. All six groups overlapped extensively, and none of them represent a specific colony. (DOCX 16 kb)

Additional file 5: **Figure S5.** Skyline plot mtDNA (HVRI) for chinstrap penguins. (JPG 144 kb)

Additional file 6: **Figure S6.** Chinstrap penguins south of their traditional breeding range. A) Breeding pairs at Waterboat Point (Gabriel González Videla base; 64°49’S, 62°51’W), B) a single individual surrounded by gentoo colonies on Doumer Island (Yelcho base; 64°65’S, 63°35’W) and c) another single bird on Avian Island (67°46’S, 68°54’W) in the midst of Adélie penguins. (JPG 43 kb)

Additional file 7: **Table S1.** Allelic richness (A), expected (H_E_) and observed heterozigosity (H_O_) values for 11 microsatellite *loci*, for all examined populations. Study site (collection location) abbreviations correspond to EI: Elephant Island, PI: Penguin Island, BP: Barton Peninsula, AI: Ardley Island, GI: Greenwich Island, MB: Miers Bluff, HP: Hannah Point, CS: Cape Shirreff, BH: Baily Head, VC: Vapour Col, KI: Kopaitic Island, GP: Georges Point, and BI: Bouvetøya. (JPG 424 kb)

Additional file 8: **Table S2.** Summary of pairwise genetic differences (*Φ*_*ST*_*)* between chinstrap penguin colonies for mtDNA marker (HVRI). Bellow the diagonal are *Φ*_*ST*_ values, and their corresponding *p*-values above the diagonal. (TIFF 2112 kb)

Additional file 9: **Table S3.** Summary of pairwise genetic differences (*F*_*ST*_*)* between chinstrap penguin colonies for mtDNA marker (HVRI). Below the diagonal are F_*ST*_ values, and their corresponding p-values above the diagonal. (DOCX 18 kb)

Additional file 10: **Table S4.** Summary of pairwise genetic differences between chinstrap penguin colonies (*F*_*ST*_*)* calculated from the 11 microsatellite *loci*. Below the diagonal are F_*ST*_ values, and corresponding p-values above the diagonal. (JPG 76 kb)

Additional file 11: **Table S5.** GeneClass2 percentage test results using microsatellite data for chinstrap penguins from 13 colonies for (a) genetic assignment using Paetkau et al. (1995) criterion and (b) first-generation migrant. Lines indicate the samples’ site collection and columns indicate the colonies to which the individuals were assigned. Colony self-assignments are in bold. (JPG 5437 kb)

## Abbreviations

∏: Average number of differences between pairs of sequences
A: Mean number of alleles per *locus*
AI: Ardley Island
AP: Antarctic Peninsula
BAPS: Bayesian Analysis of Population Structure
BH: Baily Head
BI: Bouvetøya
bp: base pair
CHD1: Chromosome-helicase-DNA binding protein
CS: Cape Shirreff
D: Tajima’*D* test
DAPC: Discriminant Analysis of Principal Components
EI: Elephant Island
FDR: False Discovery Rate
F_*IS*_: Inbreeding coefficient
Fs: Fu’s Fs test
F_*ST*_: Fixation index
GI: Greenwich Island
GP: Georges Point
h: Number of haplotypes
Hd: Haplotype diversity
He: Expected heterozigosity
HKY + G + I: Hasegawa-Kishino-Yano + gamma + invariant sites
Ho: Observed heterozigosity
HP: Hannah Point
HVRI: Hypervariable Region I
HWE: Hardy-Weinberg equilibrium
K2 + G + I: Kimura 2-parameter + gamma + invariable sites
KI: Kopaitic Island
LGM: Last Glacial Maximum
*mAIc*: mean Assignment Index
MB: Miers Bluff
MCMC: Markov Chain Monte Carlo
mtDNA: mitochondrial DNA
N: Sample size
NGS: Next Generation Sequencing
PI: Penguin Island
*r*: Relatedness between individuals
S: Polymorphic sites
SAI: Sub-Antarctic Islands
SSI: South Shetland Islands
*vAIc*: variance of Assignment Indices
VC: Vapour Col (VC)
WAP: Western Antarctic Peninsula
π: Nucleotide diversity
