# Correction to: Chinstrap penguin population genetic structure: one or more populations along the Southern Ocean?

**DOI:** 10.1186/s12862-018-1231-0

**Published:** 2018-07-25

**Authors:** Isidora Mura-Jornet, Carolina Pimentel, Gisele P. M. Dantas, Maria Virginia Petry, Daniel González-Acuña, Andrés Barbosa, Andrew D. Lowther, Kit M. Kovacs, Elie Poulin, Juliana A. Vianna

**Affiliations:** 1 Departamento de Ecosistemas y Medio Ambiente, Facultad de Agronomía e Ingeniería Forestal, Pontificia Universidad Católica de Chile, Av. Vicuña Mackenna 4860, Macul, Santiago Chile; 2 Instituto de Ecología y Biodiversidad, Departamento de Ciencias Ecológicas, Facultad de Ciencias, Universidad de Chile, Las Palmeras 3425, Ñuñoa, Santiago Chile; 3 Pontifícia Universidade Católica de Minas Gerais, PPG in Biology of Vertebrate Av, Dom Jose Gaspar, 500, prédio 41, Belo Horizonte, 30535901 Brazil; 4 Laboratório de Ornitologia e Animais Marinhos, Universidade do Vale do Rio dos Sinos, Av. Unisinos, São Leopoldo, RS 950 Brazil; 5 Departamento de Ciencias Pecuarias, Facultad de Ciencias Veterinarias, Universidad de Concepción, Av. Vicente Méndez 595, 3780000 Chillán, CP Chile; 6 Departamento de Ecología Evolutiva, Museo Nacional de Ciencias Naturales, CSIC, C/José Gutiérrez Abascal, 2, 28006 Madrid, Spain; 7 Norwegian Polar Institute, Hjalmar Johansensgata, Tromsø, Norway; 8 Departamento de Ecosistemas y Medio Ambiente, Facultad de Agronomía e Ingeniería Forestal, Pontificia Universidad Católica de Chile, Av. Vicuña Mackenna 4860, Macul, Santiago Chile

## Correction to: BMC Evolutionary Biology (2018) 18:90 10.1186/s12862-018-1207-0

Table 3 of this original publication contained some errors. The updated Table 3 is published in this correction article.

**Table 3:** Sex-biased dispersal in chinstrap penguins

	N	*F*_*IS*_	*F*_*ST*_	Assignment indices		
				Relatedness	Mean	Variance
Females	93	0.002	0.002	0.145	-0.509	9.56
Males	103	0.042	0.097	0.171	0.459	10.43
*p*-value		0.111	0.291	0.397	**0.029**	0.70

Bold values are significantly different from zero after the FDR correction (*p* < 0.05)

One-tailed test results, their corresponding *p*-values and the number (N) of females and males used for the analyses
