# Supplementary material for: Chinstrap penguin population genetic structure: one or more populations along the Southern Ocean?
Source: BMC Evol Biol. 2018 Jun 13;18:90. doi: 10.1186/s12862-018-1207-0 (PMC6001010; doi:10.1186/s12862-018-1207-0)
Supplement: Supplementary file 1 — Figure S1. Plot of assignment probabilities from STRUCTURE. A vertical bar represents an individual, and colors represent the different clusters found. All plots were generated via running 10 replicates. Figures show the optimal number of clusters for no admixture model, with independent allele frequency and no location information supplied using A) Posterior probability of K (LnP(D)) and B) Evanno’s method. (DOCX 20 kb) [file 12862_2018_1207_MOESM1_ESM.docx]

**Supplementary Material**

Chinstrap penguin population genetic structure: one or more populations along the Southern Ocean?

Isidora Mura-Jornet^1^, Carolina Pimentel^2^, Gisele PM Dantas^3^, Maria Virginia Petry^4^, Daniel González-Acuña^5^, Andrés Barbosa^6^, Andrew D. Lowther^7^, Kit M. Kovacs^7^, Elie Poulin^2^, Juliana A. Vianna^1^

1 Pontificia Universidad Católica de Chile, Departamento de Ecosistemas y Medio Ambiente, Vicuña Mackenna 4860, Macul, Santiago, Chile. imura@uc.cl; jvianna@uc.cl

2 Universidad de Chile, Departamento de Ciencias Ecológicas, Facultad de Ciencias, Las Palmeras 3425, Ñuñoa, Santiago, Chile. caropiga@gmail.com; epoulin@uchile.cl

3 Pontifícia Universidade Católica de Minas Gerais, PPG in Biology of Vertebrate Av. Dom Jose Gaspar, 500, prédio 41, Belo Horizonte, Brazil. dantasgpm@gmail.com

4 Universidade do Valle do Rio Sinos, Laboratório de Ornitologia e Animais Marinhos, Av. Unisinos, 950, São Leopoldo, RS, Brazil. mavipetry@gmail.com

5 Universidad de Concepción, Departamento de Ciencias Pecuarias, Facultad de Ciencias Veterinarias, Av. Vicente Méndez 595, CP 3780000, Chillán, Chile. danigonz@udec.cl

6 Museo Nacional de Ciencias Naturales, Departamento de Ecología Evolutiva, CSIC, C/José Gutiérrez Abascal, 2, 28006, Madrid, Spain. barbosa@mncn.csic.es

7 Norwegian Polar Institute, Hjalmar Johansensgata, Tromsø, Norway. andrew.lowther@npolar.no; kit.kovacs@npolar.no

Corresponding author:

Juliana A. Vianna, Departamento de Ecosistemas y Medio Ambiente, Facultad de Agronomía e Ingeniería Forestal, Pontificia Universidad Católica de Chile. Av. Vicuña Mackenna 4860, Santiago, Chile, Fax: 56-2-26865982, Phone: 56-2-3547210, [jvianna@uc.cl](mailto:jvianna@uc.cl)

**Table S1.** Allelic richness (A), expected (H_E_) and observed heterozigosity (H_O_) values for 11 microsatellite *loci*, for all examined populations. Study site (collection location) abbreviations correspond to EI: Elephant Island, PI: Penguin Island, BP: Barton Peninsula, AI: Ardley Island, GI: Greenwich Island, MB: Miers Bluff, HP: Hannah Point, CS: Cape Shirreff, BH: Baily Head, VC: Vapour Col, KI: Kopaitic Island, GP: Georges Point, and BI: Bouvetøya.

| ***Locus*** | **EI** | **PI** | **BP** | **AI** | **GI** | **MB** | **HP** | **CS** | **BH** | **VC** | **KI** | **GP** | **BI** | **Total** |
| --- | --- | --- | --- | --- | --- | --- | --- | --- | --- | --- | --- | --- | --- | --- |
| **AP-19** |  |  |  |  |  |  |  |  |  |  |  |  |  |  |
| A | 5 | 8 | 5 | 5 | 5 | 6 | 7 | 7 | 6 | 6 | 7 | 7 | 7 | 9 |
| H_E_ | 0.74 | 0.83 | 0.78 | 0.73 | 0.79 | 0.80 | 0.77 | 0.79 | 0.78 | 0.75 | 0.81 | 0.81 | 0.83 |  |
| H_O_ | 0.93 | 0.94 | 0.82 | 0.71 | 0.50 | 0.72 | 0.84 | 0.82 | 0.78 | 0.67 | 0.56 | 0.80 | 1.00 |  |
| **AP-26** |  |  |  |  |  |  |  |  |  |  |  |  |  |  |
| A | 7 | 8 | 10 | 6 | 6 | 8 | 7 | 6 | 6 | 8 | 8 | 8 | 8 | 11 |
| H_E_ | 0.83 | 0.81 | 0.81 | 0.79 | 0.80 | 0.88 | 0.82 | 0.80 | 0.84 | 0.82 | 0.78 | 0.85 | 0.81 |  |
| H_O_ | 0.63 | 084 | 0.69 | 0.71 | 0.69 | 0.73 | 0.80 | 0.80 | 1.00 | 0.87 | 0.92 | 0.73 | 0.71 |  |
| **AP-3** |  |  |  |  |  |  |  |  |  |  |  |  |  |  |
| A | 3 | 2 | 2 | 2 | 2 | 2 | 3 | 2 | 1 | 2 | 2 | 2 | 2 | 3 |
| H_E_ | 0.22 | 0.10 | 0.03 | 0.14 | 0.08 | 0.09 | 0.16 | 0.06 | - | 0.12 | 0.10 | 0.13 | 0.16 |  |
| H_O_ | 0.24 | 0.10 | 0.03 | 0.00 | 0.08 | 0.09 | 0.17 | 0.06 | - | 0.13 | 0.11 | 0.13 | 0.17 |  |
| **AP-90** |  |  |  |  |  |  |  |  |  |  |  |  |  |  |
| A | 8 | 7 | 9 | 9 | 8 | 4 | 9 | 9 | 7 | 8 | 9 | 9 | 8 | 9 |
| H_E_ | 0.83 | 0.80 | 0.85 | 0.91 | 0.87 | 0.77 | 0.85 | 0.84 | 0.88 | 0.89 | 0.87 | 0.89 | 0.86 |  |
| H_O_ | 0.93 | 0.87 | 0.79 | 0.82 | 0.91 | 1.00 | 0.84 | 0.90 | 0.89 | 0.80 | 0.85 | 0.86 | 0.87 |  |
| **AP-85** |  |  |  |  |  |  |  |  |  |  |  |  |  |  |
| A | 5 | 6 | 6 | 4 | 6 | 6 | 6 | 7 | 5 | 5 | 7 | 6 | 6 | 8 |
| H_E_ | 0.74 | 0.75 | 0.77 | 0.74 | 0.82 | 0.77 | 0.78 | 0.76 | 0.82 | 0.77 | 0.77 | 0.71 | 0.76 |  |
| H_O_ | 0.69 | 0.76 | 0.69 | 0.57 | 0.83 | 0.72 | 0.72 | 0.70 | 0.67 | 0.80 | 0.70 | 0.80 | 0.90 |  |
| **AP-78** |  |  |  |  |  |  |  |  |  |  |  |  |  |  |
| A | 4 | 6 | 5 | 4 | 4 | 2 | 3 | 4 | 5 | 4 | 4 | 4 | 5 | 10 |
| H_E_ | 0.36 | 0.53 | 0.25 | 0.38 | 0.27 | 0.09 | 0.28 | 0.38 | 0.55 | 0.49 | 0.29 | 0.25 | 0.44 |  |
| H_O_ | 0.41 | 0.44 | 0.17 | 0.29 | 0.14 | 0.09 | 0.28 | 0.23 | 0.67 | 0.53 | 0.26 | 0.20 | 0.39 |  |
| **CP-6** |  |  |  |  |  |  |  |  |  |  |  |  |  |  |
| A | 4 | 5 | 3 | 4 | 3 | 3 | 3 | 3 | 3 | 3 | 4 | 4 | 3 | 6 |
| H_E_ | 0.57 | 0.63 | 0.64 | 0.67 | 0.58 | 0.58 | 0.61 | 0.52 | 0.57 | 0.39 | 0.64 | 0.64 | 0.66 |  |
| H_O_ | 0.55 | 0.78 | 0.56 | 0.69 | 0.75 | 0.75 | 0.60 | 0.60 | 0.45 | 0.47 | 0.62 | 0.67 | 0.50 |  |
| **AP-61** |  |  |  |  |  |  |  |  |  |  |  |  |  |  |
| A | 6 | 6 | 7 | 5 | 5 | 6 | 7 | 8 | 6 | 7 | 7 | 6 | 7 | 10 |
| H_E_ | 0.69 | 0.64 | 0.68 | 0.71 | 0.72 | 0.68 | 0.75 | 0.76 | 0.78 | 0.80 | 0.69 | 0.62 | 0.73 |  |
| H_O_ | 0.71 | 0.58 | 0.63 | 0.50 | 0.80 | 0.50 | 0.64 | 0.64 | 0.78 | 0.80 | 0.73 | 0.53 | 0.74 |  |
| **CP-25** |  |  |  |  |  |  |  |  |  |  |  |  |  |  |
| A | 8 | 7 | 8 | 7 | 8 | 7 | 8 | 10 | 5 | 6 | 10 | 8 | 6 | 11 |
| H_E_ | 0.84 | 0.84 | 0.85 | 0.78 | 0.84 | 0.77 | 0.78 | 0.83 | 0.78 | 0.82 | 0.82 | 0.83 | 0.67 |  |
| H_O_ | 0.85 | 0.84 | 0.83 | 0.92 | 0.79 | 0.72 | 0.75 | 0.87 | 0.67 | 0.86 | 0.86 | 0.72 | 0.68 |  |
| **GP-36** |  |  |  |  |  |  |  |  |  |  |  |  |  |  |
| A | 3 | 3 | 5 | 4 | 4 | 3 | 3 | 5 | 3 | 4 | 4 | 3 | 5 | 5 |
| H_E_ | 0.31 | 0.54 | 0.43 | 0.57 | 0.58 | 0.54 | 0.44 | 0.47 | 0.39 | 0.48 | 0.46 | 0.48 | 0.52 |  |
| H_O_ | 0.21 | 0.73 | 0.31 | 0.58 | 0.58 | 0.50 | 0.60 | 0.33 | 0.44 | 0.47 | 0.39 | 0.67 | 0.52 |  |
| **GP-15** |  |  |  |  |  |  |  |  |  |  |  |  |  |  |
| A | 8 | 7 | 8 | 8 | 6 | 8 | 9 | 10 | 5 | 7 | 8 | 7 | 7 | 12 |
| H_E_ | 0.78 | 0.79 | 0.77 | 0.81 | 0.71 | 0.82 | 0.83 | 0.81 | 0.69 | 0.81 | 0.46 | 0.81 | 0.71 |  |
| H_O_ | 0.71 | 0.73 | 0.77 | 0.75 | 0.64 | 0.82 | 0.80 | 0.77 | 0.67 | 0.93 | 0.39 | 0.93 | 0.65 |  |
