# Supplementary material for: Chinstrap penguin population genetic structure: one or more populations along the Southern Ocean?
Source: BMC Evol Biol. 2018 Jun 13;18:90. doi: 10.1186/s12862-018-1207-0 (PMC6001010; doi:10.1186/s12862-018-1207-0)
Supplement: Supplementary file 2 — Figure S2. Plot of assignment probabilities from BAPS. Vertical lines represent each individual and the color refers to clusters found through this analysis. A) spatial with both, mixture and admixture models (K = 1) and B) non-spatial admixture model (K = 7). (DOCX 16 kb) [file 12862_2018_1207_MOESM2_ESM.docx]

**Supplementary Material**

Chinstrap penguin population genetic structure: one or more populations along the Southern Ocean?

Isidora Mura-Jornet^1^, Carolina Pimentel^2^, Gisele PM Dantas^3^, Maria Virginia Petry^4^, Daniel González-Acuña^5^, Andrés Barbosa^6^, Andrew D. Lowther^7^, Kit M. Kovacs^7^, Elie Poulin^2^, Juliana A. Vianna^1^

1 Pontificia Universidad Católica de Chile, Departamento de Ecosistemas y Medio Ambiente, Vicuña Mackenna 4860, Macul, Santiago, Chile. imura@uc.cl; jvianna@uc.cl

2 Universidad de Chile, Departamento de Ciencias Ecológicas, Facultad de Ciencias, Las Palmeras 3425, Ñuñoa, Santiago, Chile. caropiga@gmail.com; epoulin@uchile.cl

3 Pontifícia Universidade Católica de Minas Gerais, PPG in Biology of Vertebrate Av. Dom Jose Gaspar, 500, prédio 41, Belo Horizonte, Brazil. dantasgpm@gmail.com

4 Universidade do Valle do Rio Sinos, Laboratório de Ornitologia e Animais Marinhos, Av. Unisinos, 950, São Leopoldo, RS, Brazil. mavipetry@gmail.com

5 Universidad de Concepción, Departamento de Ciencias Pecuarias, Facultad de Ciencias Veterinarias, Av. Vicente Méndez 595, CP 3780000, Chillán, Chile. danigonz@udec.cl

6 Museo Nacional de Ciencias Naturales, Departamento de Ecología Evolutiva, CSIC, C/José Gutiérrez Abascal, 2, 28006, Madrid, Spain. barbosa@mncn.csic.es

7 Norwegian Polar Institute, Hjalmar Johansensgata, Tromsø, Norway. andrew.lowther@npolar.no; kit.kovacs@npolar.no

Corresponding author:

Juliana A. Vianna, Departamento de Ecosistemas y Medio Ambiente, Facultad de Agronomía e Ingeniería Forestal, Pontificia Universidad Católica de Chile. Av. Vicuña Mackenna 4860, Santiago, Chile, Fax: 56-2-26865982, Phone: 56-2-3547210, [jvianna@uc.cl](mailto:jvianna@uc.cl)

**Table S2** Summary of pairwise genetic differences (*Φ_ST_)* between chinstrap penguin colonies for mtDNA marker (HVRI). Below the diagonal are *Φ_ST_* values, and their corresponding p-values above the diagonal.

|  | EI | PI | BP | AI | GI | MB | HP | CS | BH | VC | KI | GP | BI |
| --- | --- | --- | --- | --- | --- | --- | --- | --- | --- | --- | --- | --- | --- |
| EI |  | 0.166 | 0.166 | 0.249 | 0.249 | 0.309 | 0.320 | 0.320 | 0.361 | 0.361 | 0.438 | 0.438 | 0.438 |
| PI | -0.01 |  | 0.438 | 0.459 | 0.494 | 0.592 | 0.592 | 0.592 | 0.592 | 0.592 | 0.592 | 0.592 | 0.592 |
| BP | 0.005 | 0.014 |  | 0.592 | 0.592 | 0.592 | 0.592 | 0.592 | 0.592 | 0.592 | 0.592 | 0.592 | 0.592 |
| AI | 0.016 | 0.009 | 0.048 |  | 0.592 | 0.592 | 0.592 | 0.592 | 0.592 | 0.592 | 0.604 | 0.604 | 0.604 |
| GI | -0.009 | -0.029 | 0.000 | 0.014 |  | 0.617 | 0.619 | 0.631 | 0.640 | 0.661 | 0.661 | 0.662 | 0.662 |
| MB | 0.059 | 0.031 | 0.004 | 0.131 | 0.058 |  | 0.662 | 0.671 | 0.671 | 0.671 | 0.679 | 0.867 | 0.867 |
| HP | 0.028 | 0.014 | -0.017 | 0.085 | 0.012 | 0.013 |  | 0.867 | 0.875 | 0.875 | 0.875 | 0.875 | 0.875 |
| CS | 0.003 | -0.015 | -0.011 | 0.059 | -0.012 | -0.001 | 0.006 |  | 0.875 | 0.875 | 0.877 | 0.877 | 0.877 |
| BH | -0.028 | -0.051 | -0.017 | -0.003 | -0.050 | 0.005 | -0.021 | -0.014 |  | 0.877 | 0.877 | 0.885 | 0.926 |
| VC | 0.019 | 0.015 | 0.006 | 0.076 | 0.013 | 0.020 | 0.002 | 0.009 | -0.023 |  | 0.955 | 0.984 | 0.984 |
| KI | 0.009 | 0.004 | -0.007 | 0.047 | 0.001 | 0.022 | -0.022 | -0.001 | -0.019 | 0.007 |  | 0.984 | 0.984 |
| GP | 0.002 | 0.012 | -0.013 | 0.058 | 0.029 | -0.042 | -0.012 | -0.002 | -0.024 | -0.040 | -0.01 |  | 0.984 |
| BI | 0.019 | 0.009 | 0.026 | 0.021 | 0.009 | 0.049 | 0.040 | 0.008 | 0.008 | 0.034 | 0.020 | 0.017 |  |
