# Supplementary material for: Chinstrap penguin population genetic structure: one or more populations along the Southern Ocean?
Source: BMC Evol Biol. 2018 Jun 13;18:90. doi: 10.1186/s12862-018-1207-0 (PMC6001010; doi:10.1186/s12862-018-1207-0)
Supplement: Supplementary file 3 — Figure S3. Posterior probabilities of population membership from the spatial model with correlated allele frequencies’ model. Lighter colors indicate higher probabilities of population membership. Three genetic clusters were identified using GENELAND. Left: Kopaitic Island, middle: Georges Point, and right: northern WAP locations and Bouvetøya. (DOCX 16 kb) [file 12862_2018_1207_MOESM3_ESM.docx]

**Supplementary Material**

Chinstrap penguin population genetic structure: one or more populations along the Southern Ocean?

Isidora Mura-Jornet^1^, Carolina Pimentel^2^, Gisele PM Dantas^3^, Maria Virginia Petry^4^, Daniel González-Acuña^5^, Andrés Barbosa^6^, Andrew D. Lowther^7^, Kit M. Kovacs^7^, Elie Poulin^2^, Juliana A. Vianna^1^

1 Pontificia Universidad Católica de Chile, Departamento de Ecosistemas y Medio Ambiente, Vicuña Mackenna 4860, Macul, Santiago, Chile. imura@uc.cl; jvianna@uc.cl

2 Universidad de Chile, Departamento de Ciencias Ecológicas, Facultad de Ciencias, Las Palmeras 3425, Ñuñoa, Santiago, Chile. caropiga@gmail.com; epoulin@uchile.cl

3 Pontifícia Universidade Católica de Minas Gerais, PPG in Biology of Vertebrate Av. Dom Jose Gaspar, 500, prédio 41, Belo Horizonte, Brazil. dantasgpm@gmail.com

4 Universidade do Valle do Rio Sinos, Laboratório de Ornitologia e Animais Marinhos, Av. Unisinos, 950, São Leopoldo, RS, Brazil. mavipetry@gmail.com

5 Universidad de Concepción, Departamento de Ciencias Pecuarias, Facultad de Ciencias Veterinarias, Av. Vicente Méndez 595, CP 3780000, Chillán, Chile. danigonz@udec.cl

6 Museo Nacional de Ciencias Naturales, Departamento de Ecología Evolutiva, CSIC, C/José Gutiérrez Abascal, 2, 28006, Madrid, Spain. barbosa@mncn.csic.es

7 Norwegian Polar Institute, Hjalmar Johansensgata, Tromsø, Norway. andrew.lowther@npolar.no; kit.kovacs@npolar.no

Corresponding author:

Juliana A. Vianna, Departamento de Ecosistemas y Medio Ambiente, Facultad de Agronomía e Ingeniería Forestal, Pontificia Universidad Católica de Chile. Av. Vicuña Mackenna 4860, Santiago, Chile, Fax: 56-2-26865982, Phone: 56-2-3547210, [jvianna@uc.cl](mailto:jvianna@uc.cl)

**Table S3** Summary of pairwise genetic differences (*F_ST_)* between chinstrap penguin colonies for mtDNA marker (HVRI). Below the diagonal are F*_ST_* values, and their corresponding p-values above the diagonal.

|  | EI | PI | BP | AI | GI | MB | HP | CS | BH | VC | KI | GP | BI |
| --- | --- | --- | --- | --- | --- | --- | --- | --- | --- | --- | --- | --- | --- |
| EI |  | 1.00 | 1.00 | 1.00 | 1.00 | 1.00 | 1.00 | 1.00 | 1.00 | 1.00 | 1.00 | 1.00 | 1.00 |
| PI | -0.009 |  | 1.00 | 1.00 | 1.00 | 1.00 | 1.00 | 1.00 | 1.00 | 1.00 | 1.00 | 1.00 | 1.00 |
| BP | -0.014 | -0.007 |  | 1.00 | 1.00 | 1.00 | 1.00 | 1.00 | 1.00 | 1.00 | 1.00 | 1.00 | 1.00 |
| AI | 0.014 | -0.003 | 0.009 |  | 1.00 | 1.00 | 1.00 | 1.00 | 1.00 | 1.00 | 1.00 | 1.00 | 1.00 |
| GI | -0.004 | -0.008 | -0.01 | -0.010 |  | 1.00 | 1.00 | 1.00 | 1.00 | 1.00 | 1.00 | 1.00 | 1.00 |
| MB | -0.021 | -0.008 | -0.019 | 0.011 | 0.001 |  | 1.00 | 1.00 | 1.00 | 1.00 | 1.00 | 1.00 | 1.00 |
| HP | -0.005 | 0.003 | -0.007 | 0.023 | 0.014 | -0.018 |  | 1.00 | 1.00 | 1.00 | 1.00 | 1.00 | 1.00 |
| CS | -0.007 | -0.006 | -0.010 | 0.023 | -0.006 | -0.017 | 0.003 |  | 1.00 | 1.00 | 1.00 | 1.00 | 1.00 |
| BH | -0.008 | -0.024 | -0.009 | 0.002 | -0.008 | -0.041 | -0.015 | -0.019 |  | 1.00 | 1.00 | 1.00 | 1.00 |
| VC | -0.004 | -0.002 | -0.020 | 0.002 | 0.000 | -0.013 | -0.003 | -0.002 | -0.002 |  | 1.00 | 1.00 | 1.00 |
| KI | 0.007 | -0.001 | -0.001 | 0.002 | -0.006 | -0.004 | 0.012 | 0.001 | -0.003 | -0.006 |  | 1.00 | 1.00 |
| GP | -0.007 | -0.001 | -0.006 | 0.016 | 0.004 | -0.011 | 0.003 | -0.009 | -0.010 | -0.017 | -0.00 |  | 1.00 |
| BI | -0.015 | -0.005 | -0.013 | 0.003 | -0.002 | -0.018 | -0.003 | -0.000 | -0.004 | -0.011 | -0.004 | -0.00 |  |
