# Supplementary material for: Chinstrap penguin population genetic structure: one or more populations along the Southern Ocean?
Source: BMC Evol Biol. 2018 Jun 13;18:90. doi: 10.1186/s12862-018-1207-0 (PMC6001010; doi:10.1186/s12862-018-1207-0)
Supplement: Supplementary file 4 — Figure S4. Discriminant Analysis of Principal Components (DAPC). The six genetic clusters identified by Adegenet are shown in different colors. All six groups overlapped extensively, and none of them represent a specific colony. (DOCX 16 kb) [file 12862_2018_1207_MOESM4_ESM.docx]

**Supplementary Material**

Chinstrap penguin population genetic structure: one or more populations along the Southern Ocean?

Isidora Mura-Jornet^1^, Carolina Pimentel^2^, Gisele PM Dantas^3^, Maria Virginia Petry^4^, Daniel González-Acuña^5^, Andrés Barbosa^6^, Andrew D. Lowther^7^, Kit M. Kovacs^7^, Elie Poulin^2^, Juliana A. Vianna^1^

1 Pontificia Universidad Católica de Chile, Departamento de Ecosistemas y Medio Ambiente, Vicuña Mackenna 4860, Macul, Santiago, Chile. imura@uc.cl; jvianna@uc.cl

2 Universidad de Chile, Departamento de Ciencias Ecológicas, Facultad de Ciencias, Las Palmeras 3425, Ñuñoa, Santiago, Chile. caropiga@gmail.com; epoulin@uchile.cl

3 Pontifícia Universidade Católica de Minas Gerais, PPG in Biology of Vertebrate Av. Dom Jose Gaspar, 500, prédio 41, Belo Horizonte, Brazil. dantasgpm@gmail.com

4 Universidade do Valle do Rio Sinos, Laboratório de Ornitologia e Animais Marinhos, Av. Unisinos, 950, São Leopoldo, RS, Brazil. mavipetry@gmail.com

5 Universidad de Concepción, Departamento de Ciencias Pecuarias, Facultad de Ciencias Veterinarias, Av. Vicente Méndez 595, CP 3780000, Chillán, Chile. danigonz@udec.cl

6 Museo Nacional de Ciencias Naturales, Departamento de Ecología Evolutiva, CSIC, C/José Gutiérrez Abascal, 2, 28006, Madrid, Spain. barbosa@mncn.csic.es

7 Norwegian Polar Institute, Hjalmar Johansensgata, Tromsø, Norway. andrew.lowther@npolar.no; kit.kovacs@npolar.no

Corresponding author:

Juliana A. Vianna, Departamento de Ecosistemas y Medio Ambiente, Facultad de Agronomía e Ingeniería Forestal, Pontificia Universidad Católica de Chile. Av. Vicuña Mackenna 4860, Santiago, Chile, Fax: 56-2-26865982, Phone: 56-2-3547210, [jvianna@uc.cl](mailto:jvianna@uc.cl)

**Table S4** Summary of pairwise genetic differences between chinstrap penguin colonies (*F_ST_)* calculated from the 11 microsatellite *loci*. Below the diagonal are F*_ST_* values, and corresponding p-values above the diagonal.

|  | EI | PI | BP | AI | GI | MB | HP | CS | BH | VC | KI | GP | BI |
| --- | --- | --- | --- | --- | --- | --- | --- | --- | --- | --- | --- | --- | --- |
| EI |  | 0.744 | 0.765 | 0.542 | 0.669 | 0.971 | 0.266 | 0.971 | 0.200 | 0.266 | 0.266 | 0.002 | 0.971 |
| PI | -0.002 |  | 0.687 | 0.740 | 0.971 | 0.971 | 0.325 | 0.619 | 0.295 | 0.159 | 0.917 | 0.002 | 0.655 |
| BP | 0.000 | 0.001 |  | 0.372 | 1.000 | 1.000 | 0.256 | 1.000 | 0.159 | 0.147 | 0.927 | 0.000 | 0.927 |
| AI | 0.008 | 0.000 | 0.010 |  | 0.971 | 0.917 | 0.266 | 0.643 | 0.232 | 0.280 | 0.669 | 0.007 | 0.656 |
| GI | 0.003 | -0.012 | -0.021 | -0.009 |  | 1.000 | 0.971 | 1.000 | 0.677 | 0.740 | 0.971 | 0.159 | 0.971 |
| MB | -0.012 | -0.012 | -0.019 | -0.002 | -0.026 |  | 0.959 | 1.000 | 0.266 | 0.331 | 0.971 | 0.130 | 0.994 |
| HP | 0.010 | 0.007 | 0.009 | 0.013 | -0.007 | -0.006 |  | 0.303 | 0.534 | 0.847 | 0.295 | 0.303 | 0.284 |
| CS | -0.006 | 0.003 | -0.006 | 0.005 | -0.011 | -0.012 | 0.007 |  | 0.245 | 0.256 | 0.847 | 0.000 | 0.971 |
| BH | 0.025 | 0.012 | 0.025 | 0.032 | 0.003 | 0.023 | 0.006 | 0.020 |  | 0.687 | 0.256 | 0.183 | 0.256 |
| VC | 0.012 | 0.019 | 0.018 | 0.017 | 0.000 | 0.013 | -0.003 | 0.013 | -0.001 |  | 0.088 | 0.677 | 0.248 |
| KI | 0.011 | -0.004 | -0.003 | 0.003 | -0.009 | -0.012 | 0.007 | -0.001 | 0.018 | 0.025 |  | 0.000 | 0.927 |
| GP | **0.045** | **0.049** | **0.047** | **0.052** | 0.023 | 0.027 | 0.008 | **0.040** | 0.023 | 0.000 | **0.054** |  | 0.007 |
| BI | -0.007 | 0.000 | -0.003 | 0.002 | -0.011 | -0.013 | 0.007 | -0.006 | 0.016 | 0.012 | -0.004 | **0.031** |  |

Bold values are significantly different from zero after the FDR correction (p < 0.05)
