# Supplementary figures and images for: Chinstrap penguin population genetic structure: one or more populations along the Southern Ocean?
Source: BMC Evol Biol. 2018 Jun 13;18:90. doi: 10.1186/s12862-018-1207-0 (PMC6001010; doi:10.1186/s12862-018-1207-0)

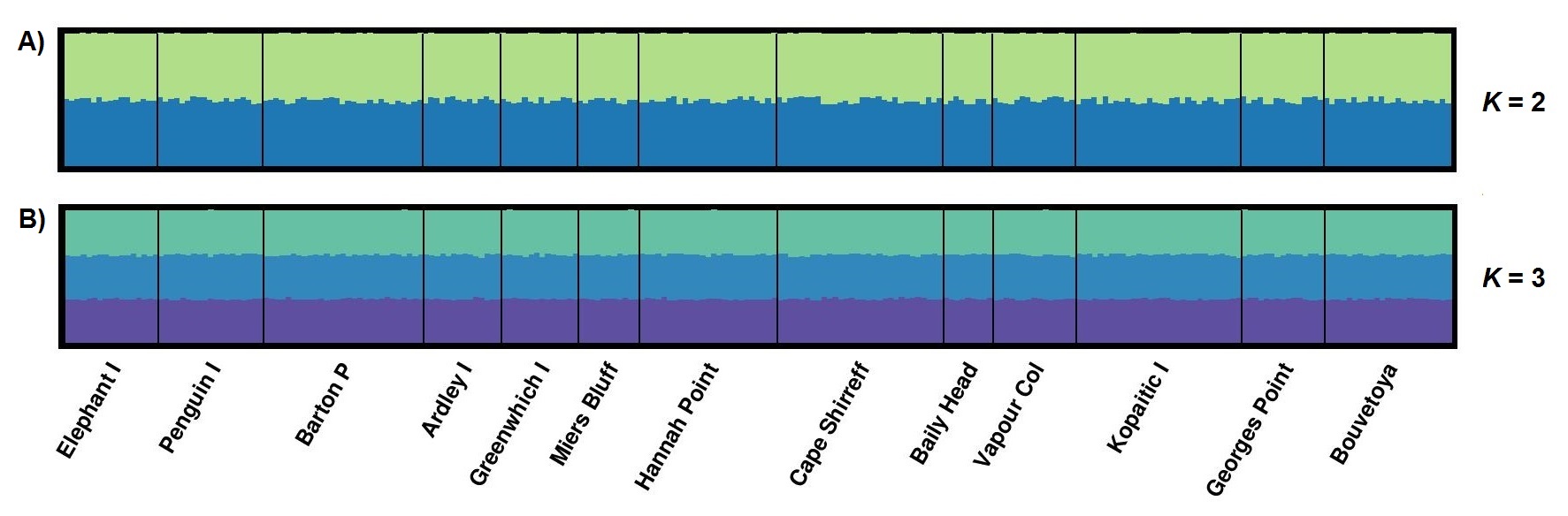

Supplement: Supplementary file 5 — Figure S5. Skyline plot mtDNA (HVRI) for chinstrap penguins. (JPG 144 kb) [file 12862_2018_1207_MOESM5_ESM.jpg]

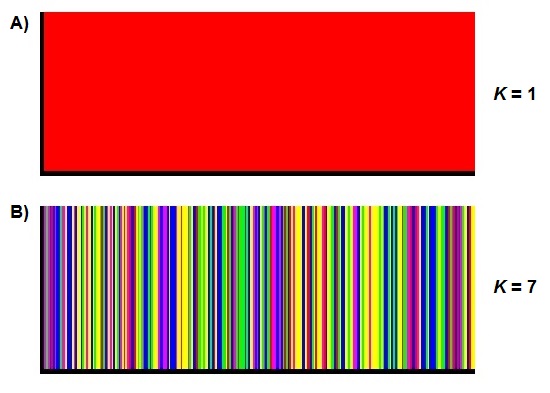

Supplement: Supplementary file 6 — Figure S6. Chinstrap penguins south of their traditional breeding range. A) Breeding pairs at Waterboat Point (Gabriel González Videla base; 64°49’S, 62°51’W), B) a single individual surrounded by gentoo colonies on Doumer Island (Yelcho base; 64°65’S, 63°35’W) and c) another single bird on Avian Island (67°46’S, 68°54’W) in the midst of Adélie penguins. (JPG 43 kb) [file 12862_2018_1207_MOESM6_ESM.jpg]

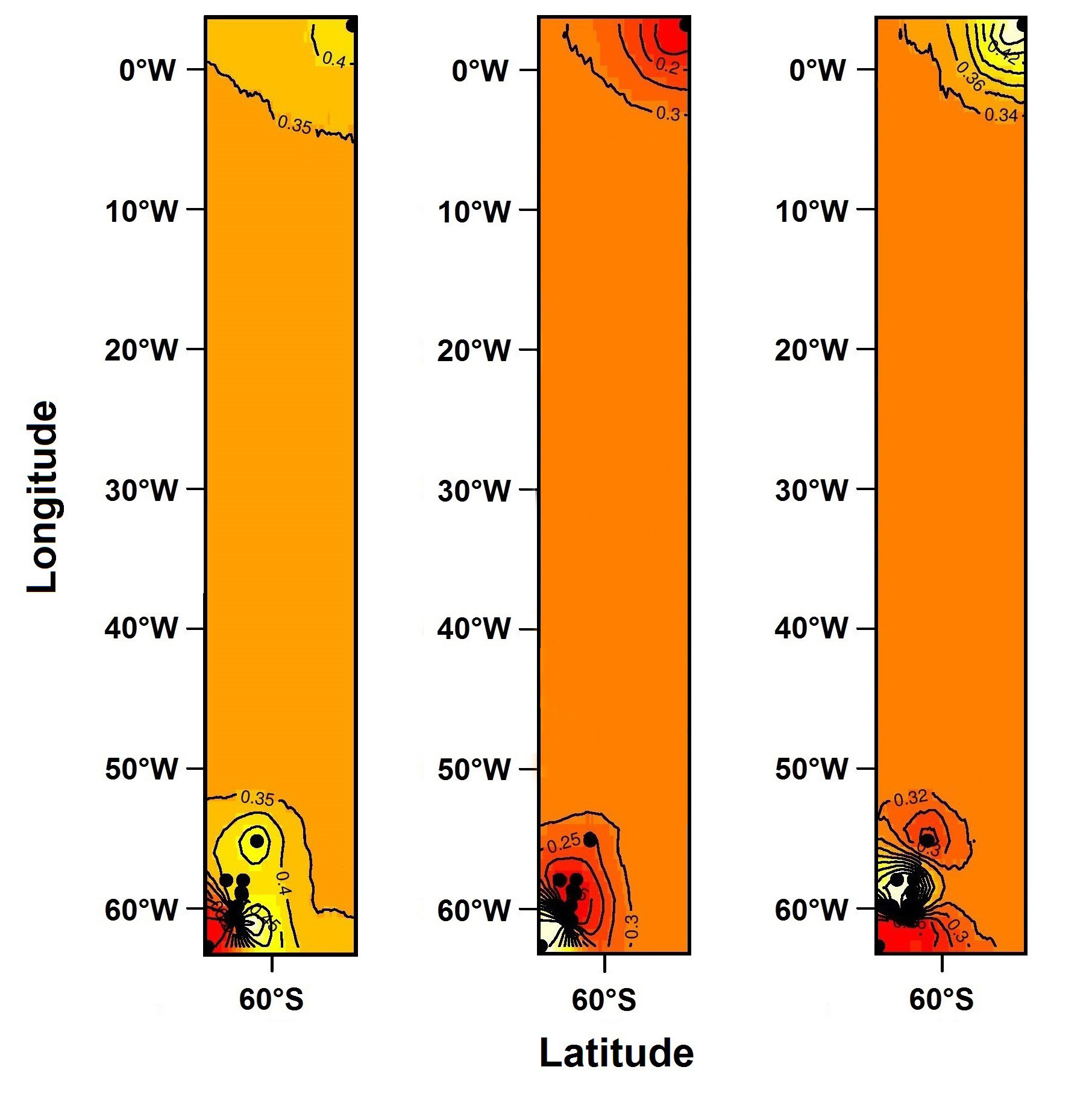

Supplement: Supplementary file 7 — Table S1. Allelic richness (A), expected (HE) and observed heterozigosity (HO) values for 11 microsatellite loci, for all examined populations. Study site (collection location) abbreviations correspond to EI: Elephant Island, PI: Penguin Island, BP: Barton Peninsula, AI: Ardley Island, GI: Greenwich Island, MB: Miers Bluff, HP: Hannah Point, CS: Cape Shirreff, BH: Baily Head, VC: Vapour Col, KI: Kopaitic Island, GP: Georges Point, and BI: Bouvetøya. (JPG 424 kb) [file 12862_2018_1207_MOESM7_ESM.jpg]

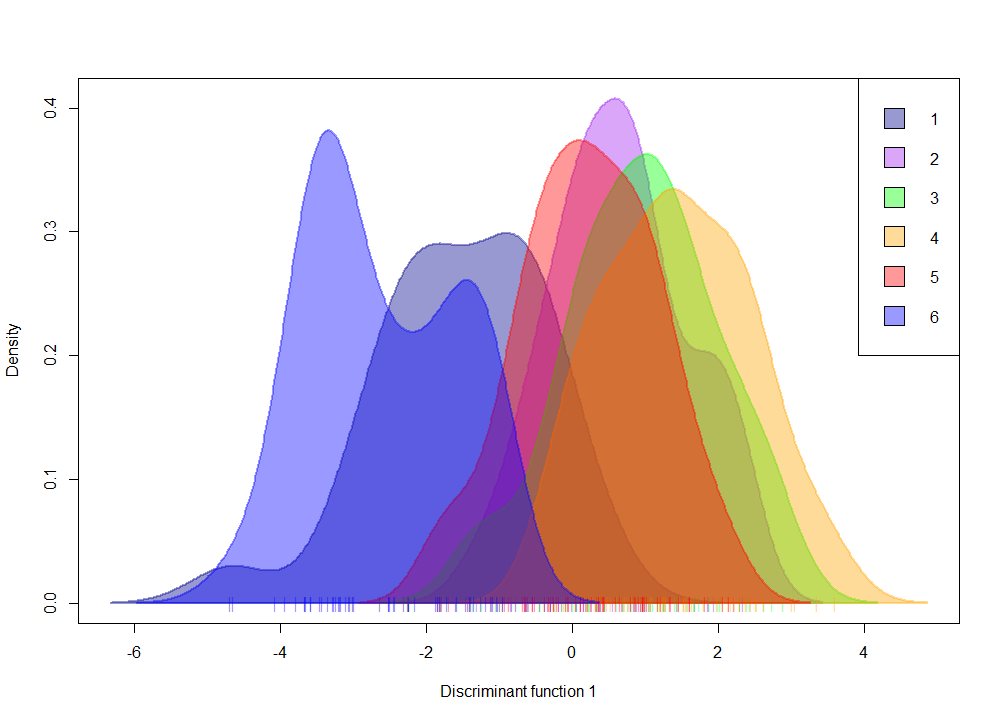

Supplement: Supplementary file 8 — Table S2. Summary of pairwise genetic differences (ΦST) between chinstrap penguin colonies for mtDNA marker (HVRI). Bellow the diagonal are ΦST values, and their corresponding p-values above the diagonal. (TIFF 2112 kb) [file 12862_2018_1207_MOESM8_ESM.tiff]

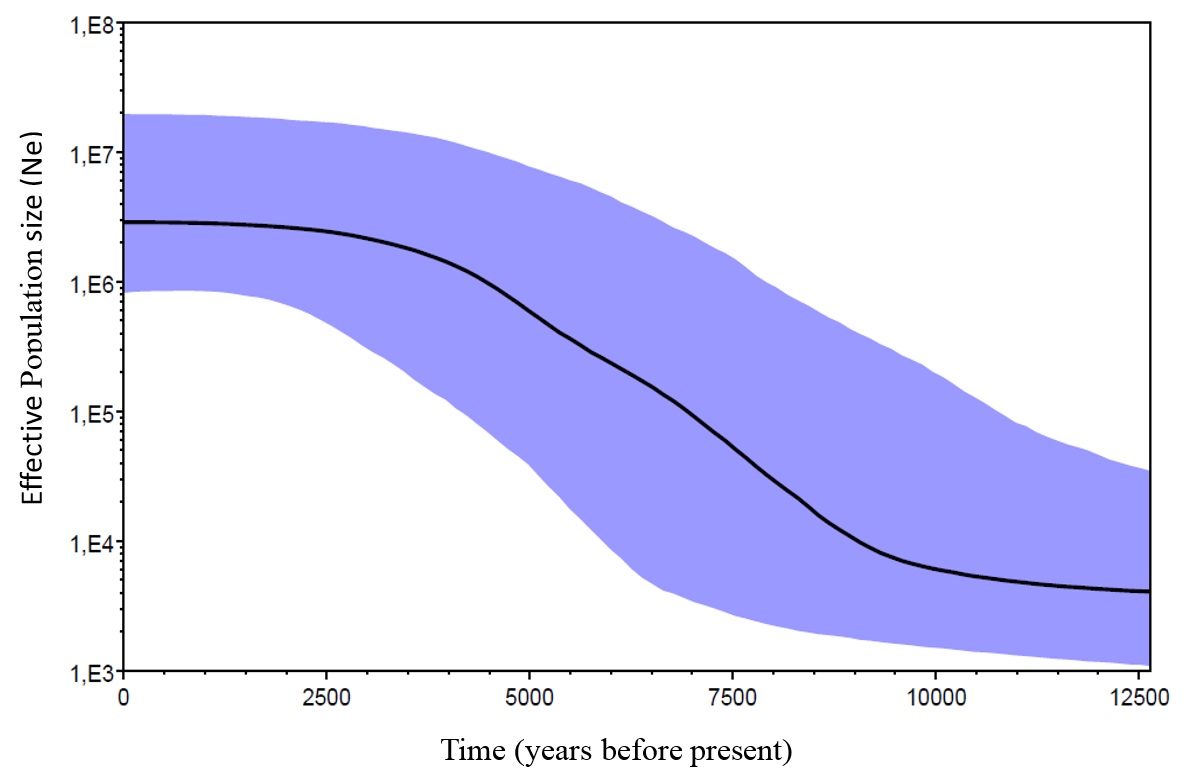

Supplement: Supplementary file 10 — Table S4. Summary of pairwise genetic differences between chinstrap penguin colonies (FST) calculated from the 11 microsatellite loci. Below the diagonal are FST values, and corresponding p-values above the diagonal. (JPG 76 kb) [file 12862_2018_1207_MOESM10_ESM.jpg]

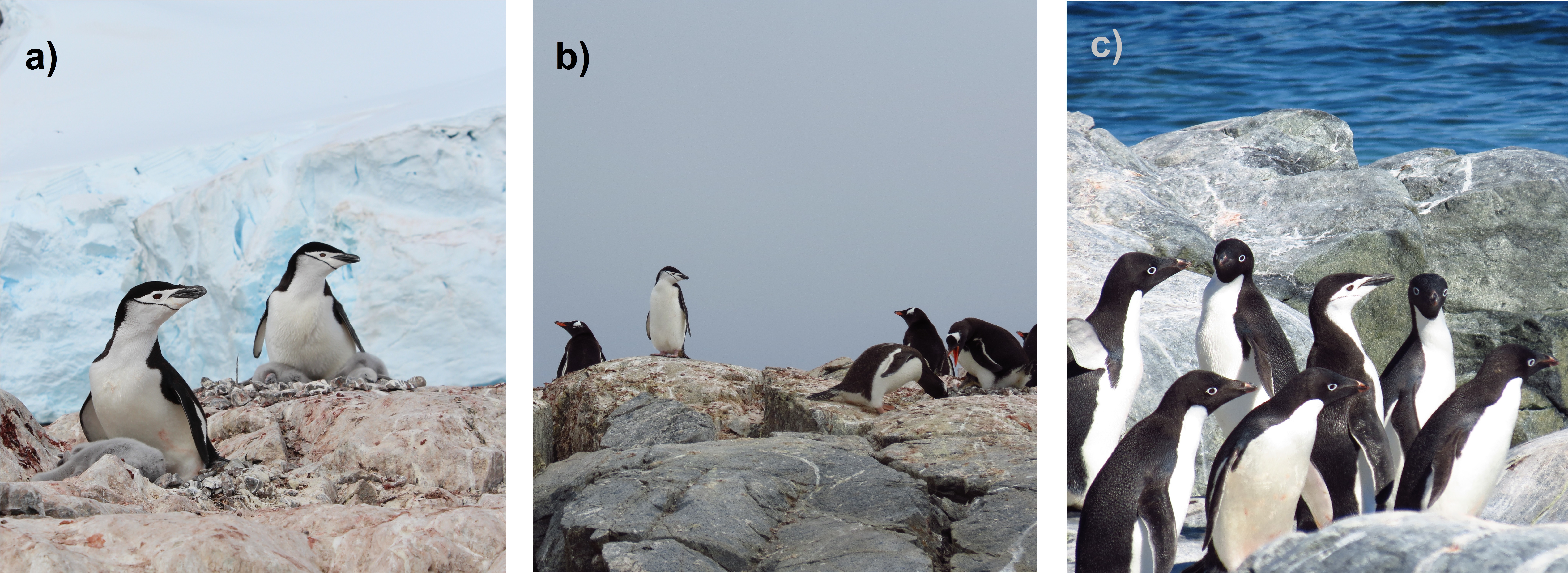

Supplement: Supplementary file 11 — Table S5. GeneClass2 percentage test results using microsatellite data for chinstrap penguins from 13 colonies for (a) genetic assignment using Paetkau et al. (1995) criterion and (b) first-generation migrant. Lines indicate the samples’ site collection and columns indicate the colonies to which the individuals were assigned. Colony self-assignments are in bold. (JPG 5437 kb) [file 12862_2018_1207_MOESM11_ESM.jpg]
