# Supplementary material for: Chinstrap penguin population genetic structure: one or more populations along the Southern Ocean?
Source: BMC Evol Biol. 2018 Jun 13;18:90. doi: 10.1186/s12862-018-1207-0 (PMC6001010; doi:10.1186/s12862-018-1207-0)
Supplement: Supplementary file 9 — Table S3. Summary of pairwise genetic differences (FST) between chinstrap penguin colonies for mtDNA marker (HVRI). Below the diagonal are FST values, and their corresponding p-values above the diagonal. (DOCX 18 kb) [file 12862_2018_1207_MOESM9_ESM.docx]

**Supplementary Material**

Chinstrap penguin population genetic structure: one or more populations along the Southern Ocean?

Isidora Mura-Jornet^1^, Carolina Pimentel^2^, Gisele PM Dantas^3^, Maria Virginia Petry^4^, Daniel González-Acuña^5^, Andrés Barbosa^6^, Andrew D. Lowther^7^, Kit M. Kovacs^7^, Elie Poulin^2^, Juliana A. Vianna^1^

1 Pontificia Universidad Católica de Chile, Departamento de Ecosistemas y Medio Ambiente, Vicuña Mackenna 4860, Macul, Santiago, Chile. imura@uc.cl; jvianna@uc.cl

2 Universidad de Chile, Departamento de Ciencias Ecológicas, Facultad de Ciencias, Las Palmeras 3425, Ñuñoa, Santiago, Chile. caropiga@gmail.com; epoulin@uchile.cl

3 Pontifícia Universidade Católica de Minas Gerais, PPG in Biology of Vertebrate Av. Dom Jose Gaspar, 500, prédio 41, Belo Horizonte, Brazil. dantasgpm@gmail.com

4 Universidade do Valle do Rio Sinos, Laboratório de Ornitologia e Animais Marinhos, Av. Unisinos, 950, São Leopoldo, RS, Brazil. mavipetry@gmail.com

5 Universidad de Concepción, Departamento de Ciencias Pecuarias, Facultad de Ciencias Veterinarias, Av. Vicente Méndez 595, CP 3780000, Chillán, Chile. danigonz@udec.cl

6 Museo Nacional de Ciencias Naturales, Departamento de Ecología Evolutiva, CSIC, C/José Gutiérrez Abascal, 2, 28006, Madrid, Spain. barbosa@mncn.csic.es

7 Norwegian Polar Institute, Hjalmar Johansensgata, Tromsø, Norway. andrew.lowther@npolar.no; kit.kovacs@npolar.no

Corresponding author:

Juliana A. Vianna, Departamento de Ecosistemas y Medio Ambiente, Facultad de Agronomía e Ingeniería Forestal, Pontificia Universidad Católica de Chile. Av. Vicuña Mackenna 4860, Santiago, Chile, Fax: 56-2-26865982, Phone: 56-2-3547210, [jvianna@uc.cl](mailto:jvianna@uc.cl)

**Table S5** GeneClass2 percentage test results using microsatellite data for chinstrap penguins from 13 colonies for (a) genetic assignment using Paetkau et al. (1995) criterion and (b) first-generation migrant. Lines indicate the samples’ site collection and columns indicate the colonies to which the individuals were assigned. Colony self-assignments are in bold.

1. Assignment test

|  | **EI** | **PI** | **BP** | **AI** | **GI** | **MB** | **HP** | **CS** | **BH** | **VC** | **KI** | **GP** | **BI** |
| --- | --- | --- | --- | --- | --- | --- | --- | --- | --- | --- | --- | --- | --- |
| **EI** | **18** | 6 | 0 | 0 | 12 | 24 | 0 | 12 | 12 | 0 | 6 | 6 | 6 |
| **PI** | 0 | **26** | 5 | 0 | 11 | 11 | 5 | 0 | 5 | 16 | 5 | 5 | 11 |
| **BP** | 7 | 3 | **10** | 0 | 24 | 7 | 7 | 17 | 3 | 0 | 3 | 3 | 10 |
| **AI** | 14 | 0 | 7 | **14** | 14 | 7 | 0 | 14 | 0 | 7 | 7 | 7 | 14 |
| **GI** | 0 | 7 | 36 | 0 | **0** | 29 | 7 | 7 | 0 | 0 | 0 | 0 | 7 |
| **MB** | 9 | 9 | 18 | 0 | 18 | **0** | 0 | 9 | 0 | 9 | 0 | 0 | 18 |
| **HP** | 4 | 12 | 12 | 0 | 16 | 12 | **8** | 0 | 4 | 16 | 8 | 8 | 0 |
| **CS** | 3 | 3 | 17 | 0 | 10 | 0 | 7 | **13** | 3 | 10 | 3 | 3 | 10 |
| **BH** | 11 | 22 | 0 | 0 | 0 | 0 | 11 | 11 | **11** | 22 | 0 | 0 | 11 |
| **VC** | 7 | 7 | 7 | 0 | 7 | 7 | 27 | 7 | 7 | **7** | 20 | 20 | 0 |
| **KI** | 10 | 3 | 13 | 3 | 20 | 10 | 3 | 7 | 7 | 0 | **0** | 0 | 3 |
| **GP** | 0 | 7 | 0 | 7 | 0 | 7 | 13 | 7 | 7 | 20 | 20 | **20** | 0 |
| **BI** | 4 | 13 | 4 | 13 | 0 | 9 | 17 | 4 | 0 | 4 | 4 | 4 | **13** |

b) First-generation migrant test

|  | **EI** | **PI** | **BP** | **AI** | **GI** | **MB** | **HP** | **CS** | **BH** | **VC** | **KI** | **GP** | **BI** |
| --- | --- | --- | --- | --- | --- | --- | --- | --- | --- | --- | --- | --- | --- |
| **EI** | **12** | 6 | 0 | 0 | 12 | 24 | 0 | 24 | 12 | 0 | 6 | 6 | 0 |
| **PI** | 0 | **21** | 5 | 0 | 5 | 16 | 5 | 0 | 11 | 21 | 11 | 0 | 5 |
| **BP** | 10 | 3 | **14** | 0 | 17 | 3 | 3 | 17 | 3 | 0 | 17 | 3 | 7 |
| **AI** | 14 | 0 | 14 | **14** | 14 | 7 | 0 | 14 | 0 | 7 | 7 | 0 | 7 |
| **GI** | 0 | 7 | 36 | 0 | **0** | 14 | 7 | 7 | 0 | 7 | 14 | 0 | 7 |
| **MB** | 0 | 9 | 18 | 0 | 18 | **0** | 9 | 9 | 0 | 9 | 9 | 0 | 18 |
| **HP** | 4 | 12 | 16 | 0 | 12 | 12 | **16** | 8 | 0 | 8 | 8 | 4 | 0 |
| **CS** | 3 | 3 | 13 | 0 | 7 | 0 | 7 | **20** | 3 | 10 | 20 | 3 | 10 |
| **BH** | 11 | 11 | 0 | 0 | 0 | 0 | 22 | 11 | **11** | 22 | 0 | 0 | 11 |
| **VC** | 7 | 13 | 7 | 0 | 7 | 0 | 20 | 7 | 7 | **13** | 0 | 20 | 0 |
| **KI** | 7 | 10 | 10 | 7 | 17 | 10 | 3 | 3 | 10 | 0 | **20** | 0 | 3 |
| **GP** | 0 | 7 | 7 | 13 | 13 | 0 | 0 | 7 | 0 | 40 | 0 | **13** | 0 |
| **BI** | 4 | 13 | 4 | 4 | 4 | 9 | 17 | 4 | 0 | 9 | 9 | 4 | **17** |
